# Usefulness of Magnifying Endoscopy with Narrow-Band Imaging for Determining Tumor Invasion Depth in Early Gastric Cancer

**DOI:** 10.1155/2013/217695

**Published:** 2013-01-17

**Authors:** Daisuke Kikuchi, Toshiro Iizuka, Shu Hoteya, Akihiro Yamada, Tsukasa Furuhata, Satoshi Yamashita, Kaoru Domon, Masanori Nakamura, Akira Matsui, Toshifumi Mitani, Osamu Ogawa, Sumio Watanabe, Mitsuru Kaise

**Affiliations:** ^1^Department of Gastroenterology, Toranomon Hospital, 2-2-2 Toranomon, Minato-ku, Tokyo 105-8470, Japan; ^2^Department of Gastroenterology, Juntendo University, Tokyo 113-0033, Japan

## Abstract

*Backgrounds*. Magnifying endoscopy with narrow-band imaging (ME-NBI) has become essential for determining tumor margin in early gastric cancer (EGC). Here, we investigated the usefulness of ME-NBI for assessment of invasion depth in EGC. *Methods*. For 119 patients who had undergone ME-NBI and *en bloc* resection by endoscopic submucosal dissection, three physicians prospectively examined high-magnification ME-NBI images for clinical features such as presence or absence of dilated vessels (D vessels). Cases with D vessels verified by at least two physicians were assigned to group V, and others were assigned to group N. We then compared clinicopathological factors associated with the groups. *Results*. Groups V and N consisted of 18 and 101 patients, respectively. There were no significant differences in age, gender, tumor size, tumor location, gross morphology, or histological type. The percentage of submucosal cancer was 9.9% (10/101) in group N and significantly higher at 33.3% (6/18) in group V (*P* = 0.007). When the presence of D vessels was considered a diagnostic criterion for submucosal cancer, diagnostic accuracy, sensitivity, and specificity were 81.5, 37.5, and 88.3%, respectively. *Conclusions*. The results suggest that identification of D vessels using ME-NBI can assist in the assessment of invasion depth in EGC.

## 1. Introduction

 With recent advancements in endoscopic submucosal dissection (ESD) techniques, it has become possible to perform *en bloc* resection of lesions endoscopically, regardless of their size, location, or presence of scarring [1]. In early gastric cancer, depth of tumor invasion and tumor margin are important indications for endoscopic treatment. However, even for some of the lesions indicated for ESD, it is necessary to consider the risk of lymph node metastasis, and a factor associated with such risk is depth of tumor invasion. Although conventional endoscopy and endoscopic ultrasonography (EUS) have been used to assess the depth of invasion of early gastric cancer, neither is regarded as perfect.

 Tumor margin is conventionally determined using conventional endoscopy and chromoendoscopy. Magnifying endoscopy with the narrow-band imaging (ME-NBI) is a recently developed technique that has proven beneficial for determining the tumor margin, and it is now widely used in clinical practice. However, the usefulness of ME-NBI for determining the depth of tumor invasion has not yet been elucidated. ME-NBI of gastric lesions has revealed the presence of tortuous dilated vessels with a diameter clearly larger than that of the irregular microvessels that often develop within lesions. We have always believed there is a close association between depth of tumor invasion and presence of dilated vessels, and therefore, in this study we aimed to elucidate the clinicopathological significance of dilated vessels and the usefulness of ME-NBI for assessing the depth of tumor invasion.

## 2. Materials and Methods

To accurately match endoscopic images with pathological features from a series of 125 early gastric cancer patients (133 lesions) who had undergone ME-NBI and *en bloc* tumor resection at our hospital from January to December 2010, we selected 119 patients (119 lesions) who did not meet the following exclusion criteria: simultaneous resection of multiple lesions during one operation and observation of multiple lesions during one ME-NBI procedure.

**Figure 1:**
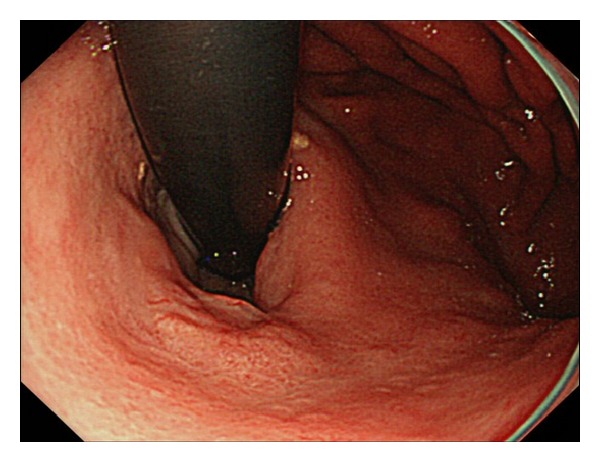
A depressed lesion of approximately 15 mm in diameter revealed by conventional endoscopy in the lesser curvature of the upper stomach.

**Figure 2:**
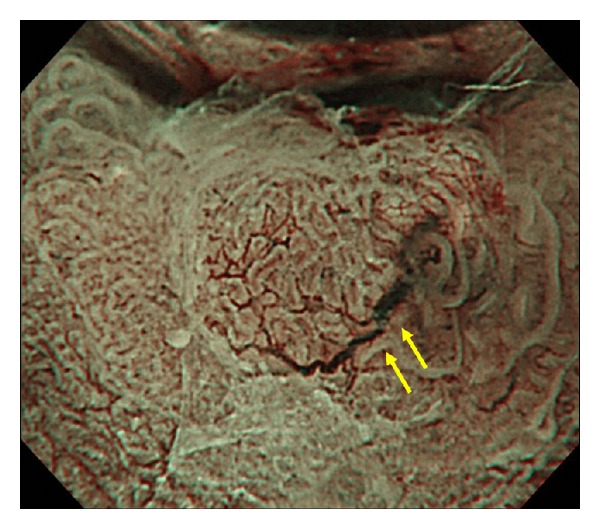
Magnifying endoscopy with narrow-band imaging (ME-NBI) image showing a clearly dilated vessel (D vessel; yellow arrows) with a diameter larger than that of nearby irregular microvessels.

**Figure 3:**
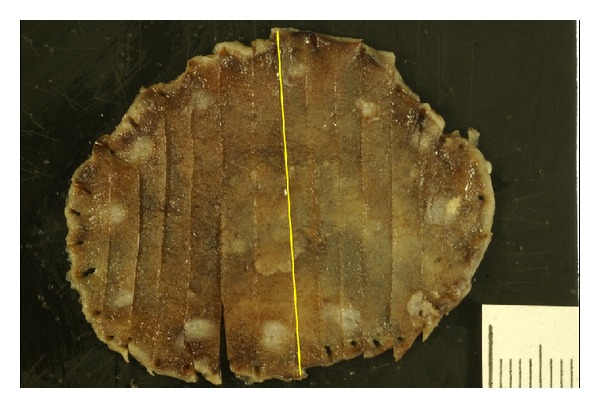
Resected specimen after endoscopic submucosal dissection.

**Figure 4:**
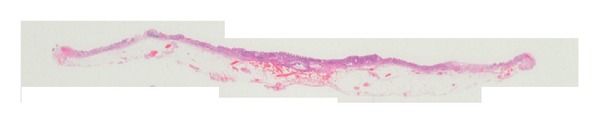
Cross-section made at the yellow line in Figure 3 and stained with hematoxylin and eosin. Pathological diagnosis was well-differentiated adenocarcinoma, 14 × 11 mm, depth sm1 (480 *μ*m), ly0, v0, and margin negative.

We then created image files consisting of high-magnification ME-NBI images and excluded conventional endoscopic images, low-magnification ME-NBI images, and EUS images. Dilated vessels (D vessels) were defined as those vessels with a diameter 3 times larger than that of the irregular microvessels that are frequently observed in the lesions (Figures 1, 2, 3, and 4). Three physicians specialized in endoscopy, who were blinded to patient information, examined the ME-NBI images for the presence of D vessels. Each physician had around 3–5 years of experience in endoscopy and 1 year in performing ME-NBI. Cases where at least two of the three physicians positively identified D vessels were assigned to the D-vessel group (group V), and others were assigned to the nonvessel group (group N). Macroscopic classification of tumors was as follows: type I (protruded), type IIa (superficial elevated), type IIb (flat), type IIc (superficial depressed), type III (excavated), and mixed type. Type I and IIa were classified as the elevated type, and the others were classified as the nonelevated type. Lesion location was classified as one of the following three regions: U (fornix, cardiac part, and upper body), M (middle body and lower body), or L (gastric angle and antrum).

The primary endpoints of the present study were diagnostic accuracy, sensitivity, and specificity of D vessels as a predictor of submucosal invasive carcinoma. In addition, various clinicopathological factors were analyzed and compared between the two groups.

### 2.1. ME-NBI Observation

 ME-NBI images were evaluated by three endoscopists (D. Kikuchi, T. Iizuka, and M. Nakamura). Pethidine hydrochloride was injected intravenously as premedication, and endoscopy was performed while monitoring blood oxygen levels. For ME-NBI, a soft transparent plastic hood (TOP Co., Tokyo, Japan) was mounted on the tip of the endoscope (GIF H260Z; Olympus Co., Tokyo, Japan) to maintain an appropriate distance from the lesion. Lesion boundaries were determined and photographed, followed initially by ME-NBI observation at low magnification and later by higher magnifications that were gradually increased. After observing the surrounding areas, we examined and photographed the center of the lesion.

### 2.2. ESD Procedure

During ESD, a Dual Knife (Olympus) and a 2-channel M scope (Olympus) or a Q260J (Olympus) were used in all patients, who were sedated with pethidine hydrochloride and diazepam. Marking dots were placed on the noncancerous mucosa approximately 5 mm from the tumor margin to provide a safety margin. A circumferential mucosal incision was performed 2 mm outside the marking dots. All lesions in this study were resected *en bloc*. 

### 2.3. Pathological Analysis

Pathological examination of the resected specimens was performed in parallel, 2 mm thick sections stained with hematoxylin and eosin. Pathological examination was done according to the Japanese classification of gastric carcinoma [2]. Tumor differentiation was classified as differentiated type (well or moderately differentiated adenocarcinoma or papillary adenocarcinoma) or undifferentiated type (poorly differentiated adenocarcinoma, signet-ring cell carcinoma, or mucinous cell carcinoma). Mixed type carcinoma with both differentiated and undifferentiated features was classified according to the predominant feature. The depth of submucosal invasion was classified as sm1 (penetration into the submucosal layer of <500 *μ*m from the muscularis mucosae) or sm2 (penetration into the submucosal layer of >500 *μ*m from the muscularis mucosae). 

### 2.4. Statistical Analysis

Data were analyzed using the unpaired student's *t*-test, chi-square test, or Mann-Whitney *U* test accordingly. A *P* value of <0.05 was considered statistically significant. 

## 3. Results

 Groups V and N comprised 18 and 101 patients, respectively. The two groups showed no significant differences in clinical factors such as mean age, sex, or location of lesion (Table 1). Moreover, pathological factors such as the mean diameter and gross morphology of the lesions also did not significantly differ between the groups. Because ESD patients were the target of the present study, more than 90% of lesions were differentiated carcinomas, and the percentage of differentiated carcinomas in the two groups was not significantly different. The proportion of undifferentiated-predominant mixed carcinomas was 27.8% (5/18) in group V and 20.8% (21/101) in group N, also not significantly different between the two groups. Patients with pathological scars accounted for 16.7% (3/18) of group V and 18.8% (19/101) of group N, a nonsignificant difference (Table 2). With regard to vascular invasion, there was one case of lymphatic involvement in group V, 3 cases of lymphatic involvement, and one case of positive venous invasion in group N, again a non-significant difference.

 The percentage of submucosal carcinoma cases was 9.9% (10/101) in group N and 33.3% (6/18) in group V (Table 2), demonstrating that group V had a significantly higher rate of submucosal carcinoma occurrence (*P* = 0.007). According to a breakdown of the submucosal carcinomas, there were 4 and 2 cases of sm1 and sm2, respectively, in group V and 7 and 3 cases of sm1 and sm2, respectively, in group N. When the presence of D vessels was set as a diagnostic criterion for submucosal carcinoma, the diagnostic accuracy, sensitivity, specificity, positive predictive value (PPV), and negative predictive value (NPV) were 81.5%, 37.5%, 88.3%, 33.3%, and 90.1%, respectively (Table 3). 

## 4. Discussion

 Advancements in endoscopic treatment have enabled a large number of early gastric cancers to be treated by ESD. Many factors associated with the risk of lymph node metastasis have been reported [3], and the depth of tumor invasion is considered a key factor. Therefore, accurate assessment of submucosal infiltration in early gastric cancer is a critical preoperative indication for ESD. Although the depth of tumor invasion has been evaluated using conventional endoscopy and EUS, the diagnostic yields of these approaches are still unsatisfactory [4, 5]. Because it is difficult to increase diagnostic accuracy using only a single modality, findings from different modalities may need to be evaluated in a comprehensive manner.

 NBI is an image enhancement technology that clearly visualizes fine mucosal structure and microvasculature by using 415 and 540 nm wavelengths which are in the absorption range of hemoglobin [6]. Today, this technology is becoming an indispensable modality for the diagnosis of early gastric cancer. ME-NBI is reported to be useful for determining the tumor margin in early gastric cancer [7], and it is currently in wide use. Although there are many reports on the usefulness of ME-NBI for the diagnosis of early gastric cancer, many such reports focus on the evaluation of the tumor margin or histological features of the lesions rather than on the depth of tumor invasion [8–10].

 Depth of invasion assessment with the use of ME-NBI has been reported in the colon and the esophagus. Kanao et al. observed microvasculature and pit appearance using ME-NBI of colorectal cancers and determined the depth of invasion. In fact, they classified colorectal cancers into types A–C and further divided type C into C1–C3. Cancers accompanied by irregularly dilated blood vessels were defined as C3, and Kanao et al. further reported the association between dilated blood vessels and submucosal massive carcinomas [11]. The association between microvessels and depth of tumor invasion has also been reported in superficial esophageal cancers. Using ME-NBI, Yoshida et al. classified microvessels in the esophageal region into type I–V. In particular, they defined irregularly dilated blood vessels accompanied by completely destroyed original intrapapillary capillary loops as type Vn blood vessels and reported that such Vn vessels are associated with submucosal infiltration [12]. 

 We conducted this study because of our hypothesis that a similar observation can be made in early gastric cancer. The results of the present study revealed that the rate of submucosal carcinoma was significantly high in group V patients with D vessel formation, thus demonstrating the association between dilated vessels observed by ME-NBI and the depth of tumor invasion in early gastric cancer. In particular, when the presence of D vessels was set as the diagnostic criterion for submucosal carcinoma, the specificity and NPV were high, at close to 90%. This indicates that lesions with D vessels are most likely submucosal carcinomas. The extent of the tumor margin is important to endoscopic information and thus needs to be acquired preoperatively with ME-NBI. It would be an extremely useful indication for endoscopic treatment if information in the depth of tumor invasion could be obtained at the same time that the information on tumor margin is obtained.

 We are, however, aware of a number of limitations in this study, one of which is the definition of D vessels. Although in the present study we defined D vessels as those having a diameter 3 times larger than that of irregular microvessels frequently observed in the lesions, there is undeniably a number of subjective aspects to this definition. The fact that an irregular microvessel, used for comparison, is one form of vascular tumor with certain variation in its diameter also presents a problem: it may be necessary in future studies to measure the diameter of vessels considered to be D vessels in order to make the criterion more objective. To overcome these weaknesses in the present study, we used only ME-NBI images which were evaluated by three physicians who had been blinded to patient information, and only when at least two of the three endoscopists agreed were the vessels judged to be D vessels. However, given the difficulty in determining the presence or absence of D vessels in some cases, the diagnostic accuracy may vary depending on how these cases are handled. In future, the examination of the actual size of D vessels is thought to be necessary. Another limitation was the low sensitivity (37.5%) of D vessels as an indicator of submucosal carcinoma, suggesting that this characteristic alone cannot be used as a diagnostic criterion. It appears necessary to create more sensitive diagnostic criteria by combining current criteria with other findings such as fine mucosal patterns. The fact that the subjects in the present study were all ESD patients might have also been a limiting factor. This is because lesions indicated for surgery were relatively large, making ME-NBI of the entire lesion difficult. In this study, we were able to perform ME-NBI because we limited ESD cases to those with a relatively small tumor diameter (mean, 21.6 mm). However, this appears to have contributed to the low percentage of undifferentiated carcinomas and lesions with ulcer scars, making it difficult to analyze the relationships between their clinicopathological factors and the presence of D vessels.

 The merits of the method used in this study may be its simplicity and high concordance rate. It is simple because ME-NBI can be switched from normal white light with the press of a button. It is also simple and valuable because D vessels can be evaluated while assessing the tumor margin with ME-NBI and thus without the need for dyes or a special device like EUS. As such, the obtained information on D vessels can be used as a helpful indicator of depth of tumor invasion. In this study, the presence of D vessels was confirmed by all three endoscopists in 14 of 18 group V patients. This suggests that a blood vessel that one endoscopist thinks is dilated will most likely be considered dilated by other endoscopists, and therefore, evaluation of D vessels is likely constructive for judging the depth of tumor invasion in early gastric cancer.

## 5. Conclusions 

Identifying D vessels using ME-NBI can facilitate the assessment of depth of tumor invasion in early gastric cancer. In the future, it will be necessary to perform more objective measurement of D vessels and to determine the site of origin. It is also necessary to investigate the relationship between histological types of tumors and D vessels through a prospective study of early gastric cancers including surgical cases.

## Figures and Tables

**Table 1:** Patient characteristic of two groups.

	Group V	Group N	
No. of patients	18	101	
Mean age (years ± SD^†^)	68.5 ± 8.6	68.9 ± 9.4	N.S.
Sex (male : female)	14 : 4	81 : 20	N.S.
Mean maximum diameter of lesions (mm ± SD)	18.9 ± 10.2	22.1 ± 17.4	N.S.
Gross type (elevated type : non-elevated type)	5 : 13	31 : 70	N.S.
Location (U : M : L^‡^)	3 : 11 : 4	17 : 36 : 48	N.S.

^†^SD: standard deviation.

^‡^U: fornix, cardiac part, and upper body, M: middle body and lower body, L: gastric angle and antrum.

**Table 2:** Comparison of histopathological features of the lesions in two groups.

	Group V	Group N	
Undifferentiated cancer	16.7% (3/18)	6.9% (7/101)	N.S.
Cancer with ulcer scar	16.7% (3/18)	18.8% (19/101)	N.S.
Submucosal cancer	33.3% (6/18)	9.9% (10/101)	*P* = 0.007

**Table 3:** D vessels and depth of tumor invasion.

	Mucosal cancer	Submucosal cancer	
D vessel (−)	91	10	101
D vessel (+)	12	6	18

	103	16	119

Diagnostic accuracy: 81.5% (97/119).

Sensitivity: 37.5% (6/16).

Specificity: 88.3% (91/103).
